# The Role of the Gut Microbiota in Anorexia Nervosa in Children and Adults—Systematic Review

**DOI:** 10.3390/ijms25010041

**Published:** 2023-12-19

**Authors:** Dana-Teodora Anton-Păduraru, Felicia Trofin, Eduard Vasile Nastase, Radu Stefan Miftode, Ionela-Larisa Miftode, Mioara Florentina Trandafirescu, Elena Cojocaru, Elena Țarcă, Dana Elena Mindru, Olivia Simona Dorneanu

**Affiliations:** 1 Department of Mother and Child Medicine, “Grigore T. Popa” University of Medicine and Pharmacy, 700115 Iasi, Romania; dana.anton@umfiasi.ro (D.-T.A.-P.); mindru.dana@umfiasi.ro (D.E.M.); 2 “Sf. Maria” Children Emergency Hospital, 700309 Iasi, Romania; elena2.cojocaru@umfiasi.ro (E.C.); tarca.elena@umfiasi.ro (E.Ț.); 3 Department of Preventive Medicine and Interdisciplinarity—Microbiology, “Grigore T. Popa” University of Medicine and Pharmacy, 700115 Iasi, Romania; olivia.dorneanu@umfiasi.ro; 4 Clinical Hospital of Infectious Diseases “Sf. Parascheva”, 700116 Iasi, Romania; larisa.miftode@yahoo.com; 5 Department of Internal Medicine II—Infectious Diseases, “Grigore T. Popa” University of Medicine and Pharmacy, 700115 Iasi, Romania; 6 Department of Internal Medicine I—Cardiology, “Grigore T. Popa” University of Medicine and Pharmacy, 700115 Iasi, Romania; radu.miftode@yahoo.com; 7 “Sf. Spiridon” Clinical Hospital, 700111 Iasi, Romania; 8 Department of Morphofunctional Sciences I—Histology, “Grigore T. Popa” University of Medicine and Pharmacy, 700115 Iasi, Romania; mio.trandafirescu@umfiasi.ro; 9 Department of Morphofunctional Sciences I—Pathology, “Grigore T. Popa” University of Medicine and Pharmacy, 700115 Iasi, Romania; 10 Department of Surgery II—Pediatric Surgery, “Grigore T. Popa” University of Medicine and Pharmacy, 700115 Iasi, Romania

**Keywords:** microbiota, gut–brain axis, anorexia, children, adults

## Abstract

Among the factors incriminated in the appearance of eating disorders, intestinal microbiota has recently been implicated. Now there is evidence that the composition of gut microbiota is different in anorexia nervosa. We gathered many surveys on the changes in the profile of gut microbiota in patients with anorexia nervosa. This review comprehensively examines the contemporary experimental evidence concerning the bidirectional communication between gut microbiota and the brain. Drawing from recent breakthroughs in this area of research, we propose that the gut microbiota significantly contributes to the intricate interplay between the body and the brain, thereby contributing to overall healthy homeostasis while concurrently impacting disease risk, including anxiety and mood disorders. Particular attention is devoted to elucidating the structure and functional relevance of the gut microbiota in the context of Anorexia Nervosa.

## 1. Introduction

Eating disorders (ED) are mental illnesses that alter the patient’s lifestyle, with consequences for the entire family and with socio-economic implications, being a cause of increased morbidity, especially in adolescents and women [1,2,3].

In recent years, there has been increased interest in studying the intestinal microbiota and its relationship with various conditions such as obesity, diabetes, arthritis, and psychiatric diseases. Anorexia nervosa (AN) is a disease of unknown aetiology, a life-threatening disease, with the highest risk of mortality among psychiatric diseases, which can be associated with depression, anxiety, obsessive compulsive manifestations, and personality disorders, with consequences on the long-term evolution [4,5]. It is accompanied by endocrine manifestations, the alteration of the immune response, increased inflammation, and intestinal dysbiosis [2,5,6]. Decreased brain volume, neuro-psychic deficits, and memory impairment have also been observed [4].

### 1.1. Epidemiology

According to different studies, the prevalence of AN is variable in the world (Table 1). In 2018, global statistical data showed that AN is the third chronic disease in adolescent girls, with a high number of cases being found in schoolgirls (1–2%), female students (3.5%), and ballerinas (7%) [7]. According to Seitz et al. (2019), in Europe, the prevalence of AN in teenage girls is 1–4% [4], while in the USA in 2011, the prevalence among adolescents aged 13–18 was 0.3% [8].

**Figure 1:**
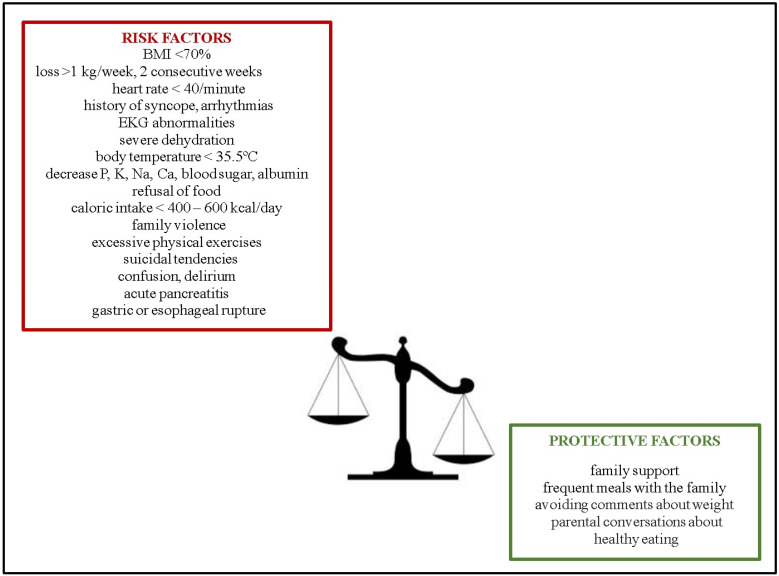
Protective and risk factors influencing AN prevalence.

The higher prevalence in different countries of the world could be explained by loss of protective factors and elevated intervention of risk factors [17,18]. The illustrative representation of protective factors is delineated in Figure 1. The onset of AN is influenced by various risk factors, as shown in Figure 1. The absence of preventive initiatives addressing body image and eating disorders constitutes an additional risk factor, as highlighted by Argyrides et al. (2020) [19]. Discrepancies in AN prevalence may also be attributed to cultural variations, comorbidities such as mental health disorders, genetic factors associated with personality traits (e.g., neuroticism and conscientiousness), alleles influencing low BMI, and variations in the oestrogen-receptor gene among females (Figure 1) [20].

### 1.2. Motivation

Adolescence is a complex and challenging period characterised by physical, psychological, and cognitive changes. It is a critical period characterised by the formation of emotional behaviours, the development of complex social behaviours, and the modification of front-limbic circuits related to neuroendocrine development [21]. Food preferences, environmental factors, and inadequate early food intake can impact the changes associated with puberty [22]. In childhood, some patients can internalize the problems that can cause the appearance of AN during adolescence, a period in which it is one of the most common chronic diseases [2,4,6].

The restriction of food intake has effects on all the systems, including the gastrointestinal tract. According to Santonicola et al. (2019), starvation determines weakness, dysfunctional musculature, reduction in absorptive surfaces, and changes in the secretion of digestive juices, with the appearance of functional or organic gastrointestinal symptoms [23].

Microbiota represents a community of microorganisms that includes bacteria, viruses, archaea, and fungi [24]. The intestinal microbiota is involved in various conditions: asthma, obesity, autism, anxiety, schizophrenia, Parkinson’s, and Alzheimer’s [25]. Just as the gut microbiota is involved in obesity, studies are showing that it is also involved in weight loss [7,26]. Presently, there is ongoing discourse concerning the link between gut microbiota and psychiatric disorders [27,28].

A microbiota is considered healthy if microorganisms with positive effects on health are more numerous [29]. There are difficulties in defining a healthy microbiota as there are over 1000 species identified in the human gut, and a person has approximately 160 of these species [30]. Intestinal dysbiosis is characterised by the loss of beneficial microorganisms (such as *Roseburia*, and *Firmicutes*), the growth of harmful species (*Clostridia*, *Enterobacteriaceae*, *Methanobrevibacter smithii*), and the reduction in the diversity of species, with the alteration of the composition of the microbiome having consequences on the state of health [31,32,33]. Dysbiosis is responsible for diseases that affect the immune response and, also, causes a decrease in the absorption of essential nutrients (amino acids, vitamins), leading to cachexia [32].

Anorexia nervosa is a multifactorial disease. At the brain level, patients with AN show an activation of the prefrontal cortex and insula—these two regions being essential for the representation of the body schema and for cognitive functions [2]. Garcia-Gil Mercedes et al. (2022) mentioned that, in AN, there is a hypoactivation in brain areas that is related to reward and interoceptive processing and a hyperactivation of areas involved in cognitive control. The bilateral interaction between the brain and the gut involves the participation of the autonomic and enteric nervous systems (ENS), the hypothalamic–pituitary–adrenal (HPA) axis and the immune system, as well as the communication between the brain and the gut microbiota. The brain–gut–microbiota axis is involved not only in gastrointestinal diseases but also in weight regulation and eating disorders [2].

### 1.3. Objectives of the Study

The objectives of our review of the microbiota in individuals with AN encompass a multifaceted array of critical goals within the realm of our investigation. These include the synthesis of knowledge, the formulation of hypotheses, the identification of discernible microbiota patterns, the exploration of clinical implications, the potential advancement of therapeutic strategies, and the facilitation of contributions to the broader scientific discourse. Furthermore, to the best of our knowledge, prior investigations in gut microbiota in patients with AN have frequently yielded heterogeneous outcomes.

## 2. Materials and Methods

### 2.1. Search Strategy

The current review includes 108 studies. The initial records were identified through a database search using “microbiota” and “anorexia nervosa”. We executed methodical searches using established databases—PubMed and Google Scholar. After the title screening, 1925 articles were excluded from consideration on account of a misalignment between their titles and the research objectives outlined in the paper. Subsequent to the abstract examination, various articles were omitted from the analytical framework based on considerations pertaining to their topical relevance, publication date, accessibility constraints, or overall pertinence to the research inquiry. Following a comprehensive examination of the complete texts, some articles were disregarded on the grounds of factors encompassing relevance, methodological incongruity, scope misalignment, issues related to quality and credibility, or language barriers. 

### 2.2. Study Selection

The process of selecting and curating articles for inclusion in our review adhered to a set of rigorous criteria. These criteria encompassed alignment with our central research question, “Is gut microbiota modified in AN?”; adherence to the research objectives; consideration of the publication year; the categorization of scientific research; and an assessment of the quality in the presentation of findings. These searches were meticulously structured, incorporating relevant keywords and phrases, and further refined through the application of Boolean operators such as “and” and “or” to enhance search precision.

The chosen studies underwent a qualitative analysis that scrutinized their adherence to principles of scholarly composition, clarity, brevity, citation frequency, sample size, the provision of pertinent data, the articulation of results, and the formulation of conclusions. These individual facets were amalgamated and incorporated into the final narrative synthesis.

## 3. Results

**Figure 2:**
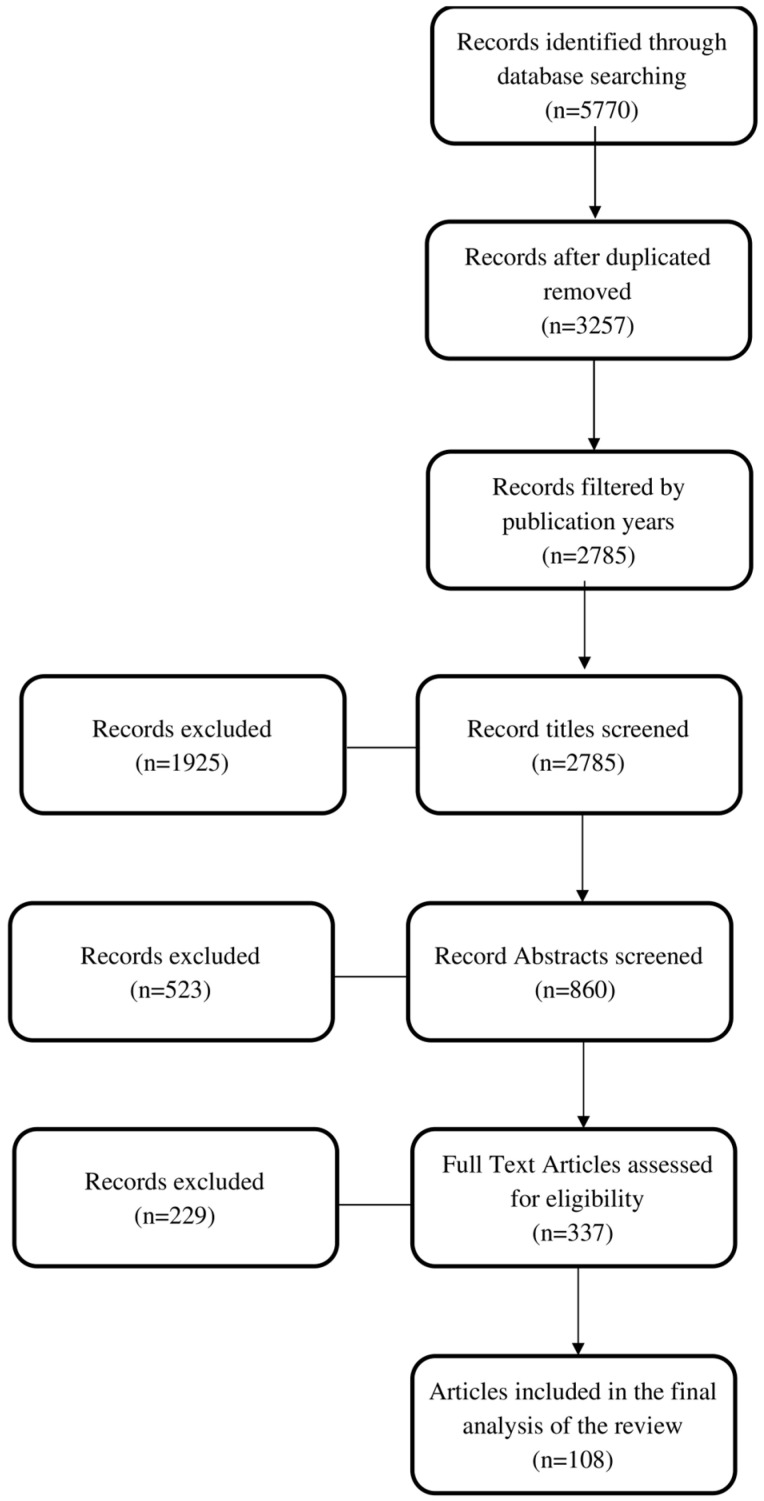
The review flowchart.

Following the data extraction phase, the information derived from the selected articles underwent a meticulous process of categorization and synthesis. A carefully structured framework was methodically constructed to underpin the development of our review article. The following flowchart (Figure 2) delineates the sequential progression of information across various stages inherent to our review process. It visually represents the tally of records ascertained, incorporated, and eliminated.

### 3.1. Influences of Gut Microbiota

Neuroscientists are increasingly recognizing the significance of the “bottom-up” influence of microbial entities themselves. Several studies have highlighted the vital role of commensal bacteria in central nervous system (CNS) function, further shedding light on the impact of these microorganisms on brain activities [27].

The stress response system undergoes functional immaturity at birth and undergoes progressive development during the postnatal period, which concurs with the establishment of intestinal bacterial colonization. Investigations involving maternal separation in rats have revealed that neonatal stress induces enduring modifications in the diversity and composition of gut microbiota. These microbial alterations could potentially contribute to the observed long-term changes in stress reactivity and stress-related behaviour in the affected rats [34].

Moreover, recent evidence from an animal model of stress-induced social disruption indicates an indirect role of microbiota in the stress response, specifically influencing some of the stress-induced changes in inflammation [35]. Stress has been recognized to heighten intestinal permeability, thereby providing an avenue for bacteria to translocate across the intestinal mucosa and directly interact with both immune cells and neuronal cells of the enteric nervous system [36]. This pathway represents a potential mechanism by which the microbiota may influence the central nervous system via the immune system and ENS under the conditions of stress.

Notably, a recent study demonstrated that the pretreatment of rats with the probiotic *Lactobacillus farciminis* effectively mitigated the increase in intestinal permeability typically resulting from restraint stress, while simultaneously preventing associated hyper-reactivity of the HPA [37]. These findings underscore the intriguing potential of the gut microbiota to modulate stress responses and related physiological processes, offering novel insights into the bidirectional communication between the gut and the brain under stress conditions. Elevated cortisol and heightened stress levels have also been shown to induce an increase in gut permeability, as evidenced in previous studies [38].

### 3.2. Gut Microbiota in AN

The complexity of the intestinal microbiome consisting of a multitude of species and its interaction with the brain makes studying the microbiota-associated behavioural effects complicated [6]. The role of the gut–brain axis in eating disorders is obvious, due to chronic caloric restriction, macronutrient deficits, and osmotic disturbances resulting in alterations of the intestinal microbiome [31]. The gut microbiota is recognized as a player with an important role in the pathophysiology and treatment of psychiatric disorders [39]. In AN there is an imbalance of the intestinal microbiome that contributes to the pathogenesis of the disease, the abnormal composition is an important factor that contributes to the cachexia present in these patients [32]. The intestinal microbiome plays an important role in the emergence of somatic and psychological manifestations of AN. The interaction between the microbiome, intestine, and brain is a risk factor for the onset and development of AN [40]. The composition of the gut microbiome is altered in AN, with dysbiosis being able to precede the onset of AN [5].

Two hypotheses have been issued regarding the role of the microbiota in AN:

1. The existence of changes in the microbiota induces and/or contributes to food restriction;
2. For each patient, the intestinal microbiota determines the type of malnutrition (marasmus or kwashiorkor) [22].

Different studies show that in AN there is a specific microbiota, in the sense that new species of *Firmicutes*, *Bacteroidetes*, and *Actinobacteria* are present in the faeces (Table 2).

Other studies have shown:

- A decrease in *Eubacterium*, *Roseburia*, *Anaerostipes*, and *Peptostreptocaccaceae* species [49].
- A reduction in *Bacteroidetes* [50].
- A decrease in *Bacteroidetes*, *Faecalibacterium*, *Agathobacter*, *Balutia*, and *Lachnospira*, an increase in *Ruminococcaceae*, *Alistipes*, and *Clostridiales* and no difference regarding fungi [51].
- An increase in *Enterobacteriaceae*, but without significant differences compared to the control group [52].
- An increased level of *Enterobacteriaceae* and *Alistipes* and a decreased level of *Faecalibacterium* [53].

Terry et al. (2022) observed an increase in *Bifidobacterium* spp. and *Odoribacter* spp. and a decrease in the level of *Haemophilus* spp. [33]. Lach et al. (2018), in studies effectuated in animals with depression, observed an increase in *Bacteroidetes* and a decrease in *Firmicutes*, while Yang et al. (2020), in human studies, found a higher abundance of *Enterobacteriaceae*, *Alistipes*, and *Bacteroidales* and a lower abundance of *Lachnospiraceae* and *Faecalibacteria* [54,55]. In humans with anxiety, Jiang et al. (2018) observed a higher abundance of *Escherichia*, *Shigella*, *Fusobacterium*, and *Ruminococcus* [56].

Microbiota changes occur in patients with AN even if they do not have severe malnutrition. The growth of *M. smithii*—a methanogenic archaea—could precede malnutrition [22]. Restrictive regimes (reduction in caloric intake by 10–40%, 10 weeks) can lead to the alteration of the composition of the microbiota with the decrease in the species *Blautia coccoides* and the increase in *Bacteroides* [57]. In humans, short-term carbohydrate restriction (24–164 g/day, 4 weeks) reduces butyrate-producing bacteria and butyrate.

In their 2023 research publication, Xia et al. established a notable association between AN and specific microbial species. Their findings indicate that certain microbial taxa, including *Actinibacteria*, *Bilophila*, *Holdemania*, *Lactobacillus*, and *Ruminococcaceae*, function as risk factors for the occurrence of AN, while others such as *Eubacteriumnodatum* and *Melaina bacteria* act as protective factors. Furthermore, the investigation into the impact of AN on the gut microbiome revealed that the abundance of *Alphaproteobacteria*, *Coriobacteriaceae*, *Christensenellaceae*, and *Anaerostipes* species is influenced by the presence of AN [58].

Anorexia nervosa affects not only bacteria but also viruses and eukaryotes. It is known that damage to the intestinal barrier and increased intestinal permeability are involved in the physiopathology of functional gastrointestinal disorders and that gut dysbiosis has a role in the regulation of gut barrier permeability. Thus, dysbiosis can contribute to the increase in intestinal barrier permeability and a low grade of inflammation and also to the onset or maintenance of functional gastrointestinal disorders associated with AN [5].

Comparative analyses indicate distinct gut microbiome compositions in healthy individuals, with discernible variations between adolescents (11–18 years) and adults aged 19 years and above. Specifically, the microbiota of individuals in these age groups is characterised by a notable abundance of species such as *Bifidobacterium* and *Clostridium*, while species such as *Prevotella* and *Sulterella* exhibit lower prevalence [21]. The examination of gut microbiota profiles across adolescence and adulthood reveals a decline in *Bifidobacteriaceae* levels with advancing age. Puberty onset is marked by an increased abundance of *Firmicutes* and *Clostridiales*, accompanied by a concomitant decrease in *Bacteroidales*, as elucidated by Calcaterra et al. in 2022 [59]. Post-pubertal development introduces significant distinctions, particularly in teenage girls, where *Ruminococcaceae* and *Lachnospiraceae* species increase, while *Bacteroidales* and *Streptococcus* decrease. Additionally, adolescents exhibit noteworthy reductions in *Lactobacillus*, *Escherichia*, and *Coriobacteriaceae*, as investigated by del Castillo-Izquierdo et al. (2022) [60].

**Figure 3:**
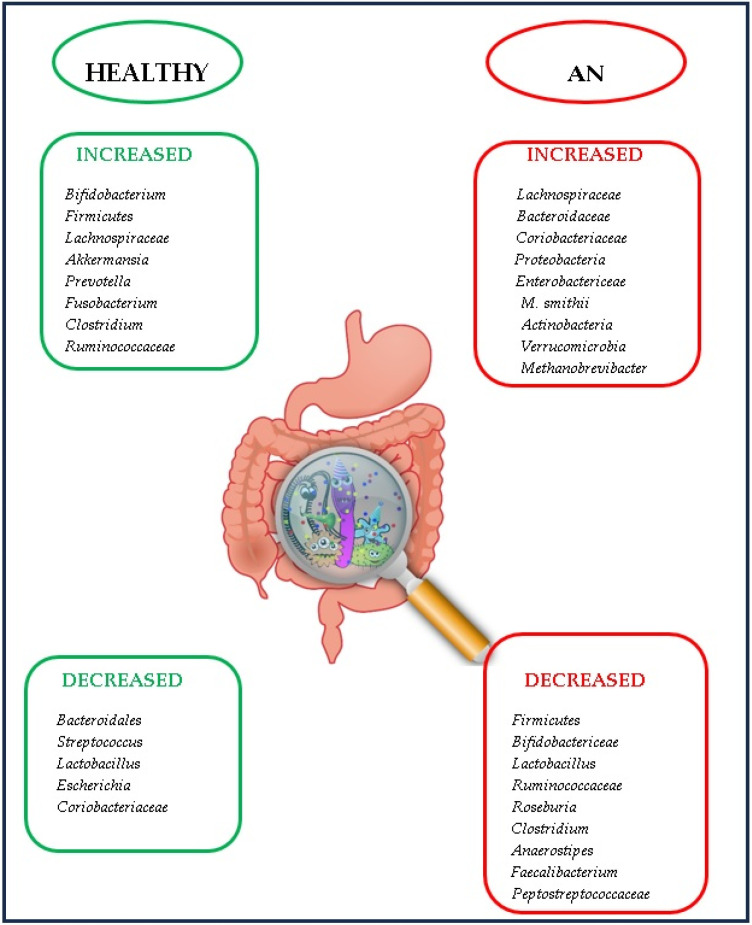
Comparison between gut microbiome in healthy individuals and in anorectic patients.

Sex-based variations in adults are evident, with women displaying higher levels of *Akkermansia* and *Ruminococcus*, and men exhibiting increased prevalence of *Prevotella* and *Fusobacterium*. These distinctions may be attributed to divergent dietary patterns, particularly the elevated consumption of animal proteins in men. A study referenced by Calcaterra et al. in 2022 revealed a heightened abundance of *Bacteroides* and *Prevotella* in healthy males compared to fertile females [59]. In the elderly, microbiota diversity diminishes, particularly concerning beneficial species (*Bifidobacterium*, *Lactobacillus*, *Faecalibacterium prausnitzii*), while the presence of pathogenic bacteria with pro-inflammatory effects increases (*E. coli*, *Enterobacter*, *Bacteroides*, *C. difficile*), as outlined by Berding et al. in 2021 (Figure 3) [30].

### 3.3. The Operational Mechanisms through Which the Gut Microbiota Functions in AN

Current investigations have demonstrated perturbations in the intestinal microbiota composition among individuals diagnosed with AN, encompassing disparities in both microbial diversity and concentration. The precise mechanistic underpinnings through which dysbiosis exerts its influence on eating disorders and neurocognitive conditions remain incompletely elucidated. One plausible mechanism involves the host’s immune system, operating within the framework of “molecular mimicry”, wherein bacteria generate compounds that emulate endogenous host molecules. Proteomic analyses have unveiled that the caseinolytic protein protease B (ClpB), emanating from commensal *Escherichia coli*, functions as an antigenic mimic of alpha-melanocyte-stimulating hormone [61]. This discovery has prompted exploration into the realm of autoantibodies targeting alpha-melanocyte-stimulating hormone, a factor integral to the regulation of food intake and behaviour, thereby opening an intriguing avenue for investigating the molecular mechanisms underpinning disordered eating [62]. Experimental studies involving mice immunized with bacterin ClpB have demonstrated diminished body weight, reduced food consumption, and ameliorated anxiety levels compared to control subjects. Furthermore, it has been observed that individuals diagnosed with AN exhibit elevated levels of plasma ClpB protein [63,64].

The prospective impacts of interventions on the gut microbiota in anorexia nervosa encompass alterations in microbiota composition, neurotransmitter production, immune system regulation, hormonal equilibrium, as well as nutrient absorption and metabolism. Interventions are designed to strategically modulate the gut microbiota composition, with the overarching objective of reinstating a more diversified and harmonized microbial milieu in individuals afflicted with anorexia nervosa. Certain interventions are formulated to specifically influence the production of neurotransmitters by the gut microbiota, notably serotonin, and gamma-aminobutyric acid (GABA), with the aim of addressing mood regulation and mental health dimensions concomitant with anorexia nervosa. Targeting the immune-modulatory attributes of the gut microbiota is another avenue of intervention, seeking to rectify any dysregulation in the immune system and mitigate chronic inflammation, which are recognized factors in psychiatric disorders, including eating disorders. Strategic initiatives may also centre on modulating the regulation of hormones associated with appetite and metabolism, exemplified by ghrelin and leptin, through interventions that influence the gut microbiota. Additionally, interventions may seek to influence nutrient absorption and energy metabolism by rectifying anomalies in the composition of the gut microbiota, thereby potentially contributing to the amelioration or prevention of anorexia nervosa [64,65].

At the molecular biology level, the mechanisms of interventions include the deployment of probiotics and prebiotics, the implementation of dietary modifications, the utilization of faecal microbiota transplantation, and the application of psychobiotics.

Probiotics, characterised as live microorganisms with discernible health benefits, and prebiotics, defined as substances fostering the proliferation of beneficial bacteria, serve as modalities to influence the composition of the gut microbiota [64,65].

Modulating the intestinal microbiota through the application of probiotics emerges as a promising approach to addressing various disorders associated with the gut–brain–microbiota axis [24]. Evidence from both human and animal studies substantiates the efficacy of probiotic treatment in alleviating anxiety [66]. The hyperactivity of the hypothalamic–pituitary–adrenal (HPA) axis can be mitigated through the administration of probiotics, specifically those containing *Lactobacillus* [28]. Gröbner et al. (2022) posit that probiotic administration in AN may serve as an adjunctive therapy, contributing to the normalization of microbiota composition and the reduction in inflammation and intestinal discomfort [67]. Notably, the administration of probiotics containing *Bifidobacteria* and *Lactobacillus* has been observed to enhance the abundance of *Roseburia* and exert positive effects during the recovery period [1]. Furthermore, the use of antidepressant therapy has been demonstrated to mitigate stress-induced alterations in the intestinal microbiota [62].

Diet is considered the most potent factor and modulator that affects the composition of gut microbiota [68]. Dietary interventions, involving the introduction of specific nutrients or dietary patterns, hold the potential to impact the configuration of the gut microbiota. The prospect of personalized nutritional interventions is explored with the objective of fostering a microbiota profile conducive to mental health [64,65].

Dietary patterns play a pivotal role in shaping the composition of the intestinal microbiota, influencing both its diversity and abundance. The intricate relationship between diet and the intestinal microbiota is bidirectional, with the microbiota serving as both a mediator of the dietary effect and a modulator of the metabolic response to the diet [30,69]. Beyond influencing species abundance, dietary constituents can also impact the kinetics of microbial populations [57]. Moreover, dietary choices may induce alterations in the intestinal microbiota, compromising the integrity of the mucous layer and facilitating the infiltration of luminal microbes into dendritic cell extensions, subsequently activating these cells. Additionally, dietary components prompt the release of immune mediators into the systemic circulation, giving rise to metabolic endotoxemia, which, in turn, triggers immune activation in various organs, including the brain [70]. The composition of the intestinal microbiota can be altered not only by dietary factors but also by diverse therapeutic interventions. Table 3 presents the results of different studies regarding the diet and gut microbiota.

The dietary profile of individuals diagnosed with AN markedly diverges from that of their healthy or normal-weight counterparts, as documented in several studies that consistently report diminished consumption of macronutrients (carbohydrates, proteins, fats), vitamins, and micronutrients, coupled with an elevated intake of dietary fibers [70,76,77]. Pertinently, specific bacteria such as *Bifidobacterium*, *Bacteroides*, and *Verrucomicrobia*, which metabolize non-digestible carbohydrates, exhibit altered dynamics in patients with AN. In the absence of these bacteria, patients resort to utilizing host-derived glycans, particularly mucins within the protective layer of the intestine, thereby heightening susceptibility to opportunistic infections [74].

Noteworthy observations by Patsalos et al. (2021) in their study, reveal reduced caloric intake, as well as diminished intake of proteins, carbohydrates, cholesterol, monounsaturated fatty acids (MUFAs), and zinc in comparison to a group of individuals who have recovered from AN. The repercussions of decreased protein consumption in AN patients manifest in the occurrence of edema and muscle atrophy. Additionally, the diminished intake of zinc, may contribute to the pathophysiology of depression in individuals with AN [78]. Dietary restrictions in AN can lead to deficiencies in essential fatty acids and polyunsaturated fatty acids, contributing to the physical and mental symptoms of the disorder [79]. Paradoxically, despite reduced caloric intake, various studies have reported hypercholesterolemia in patients with AN, attributable to compromised cholesterol and bile acid metabolism [80,81].

While the primary focus of nutritional rehabilitation in AN revolves around weight restoration and optimizing nutritional status, a thorough comprehension of the implications of current nutritional interventions on the gut microbiome necessitates the consideration of additional factors to augment treatment outcomes for AN [82].

The administration of antibiotics is associated with adverse effects on the intestinal microbiota, manifesting as reduced species diversity, altered metabolic activity, the selection of antibiotic-resistant microorganisms, and the onset of antibiotic-associated diarrhoea. Recurrent infections with *Clostridium difficile*, which produces toxins A and B, can lead to the erosion of the intestinal mucosa, as elucidated by Ramirez et al. (2020) [83]. The oral administration of antibiotics induces disruptions within the intestinal microbiome, heightening susceptibility to colonization by various intestinal pathogens, as detailed by Dahiya et al. in 2023 [84].

Investigations by Jernberg et al. in 2007 and Löfmark et al. in 2006 demonstrated that a 7-day course of Clindamycin led to a reduction in *Bacteroides* and *Enterococcus* populations [85,86]. Similarly, Rashid et al.’s 2015 study, focusing on adults undergoing a 10-day regimen of Ciprofloxacin, revealed a decreased abundance of *Bifidobacterium*. Furthermore, a decline in *Lactobacillus* and *Bifidobacterium* was observed following a 10-day administration of Clindamycin [87].

Palleja’s 2018 study, demonstrated that the administration of a combination of meropenem, gentamicin, and vancomycin in adults resulted in an increased prevalence of *Enterobacteriaceae* and a reduction in *Bifidobacterium* [88]. Similarly, Kim et al.’s investigation in 2021 reported a decrease in the abundance of *Lachospiraceae* and *Ruminococcaceae* following the use of vancomycin and imipenem [89].

Faecal microbiota transplantation, involving the transfer of faecal material from a healthy donor to a recipient, represents an investigational strategy aimed at restoring a more salubrious gut microbiota; however, its application in anorexia nervosa remains in the early stages of exploration. Psychobiotics, constituted by live bacteria ingested in sufficient quantities to confer mental health benefits, constitute interventions designed to influence the bidirectional communication between the gut and the brain [64,65].

### 3.4. Depression Linked to AN through Gut Microbiota

Studies on the link between the dysbiosis present in anorectic patients and depression have shown a negative correlation between the number of bacterial species and their level of depression [47].

Depression is associated with changes in intestinal permeability and increased systemic inflammation, as well as changes in the microbiota. During anxiety and depression, which we also encounter in AN, the gut microbiota is unbalanced. Therefore, changes in the microbial flora specific to this pathology may occur:

- The growth of *Actinobacteria* [90].
- A decrease in *Actinobacteria*, *Dialistea*, and *Coprococcus* and an increase in *Proteobacteria*, and *Alistipes* [91].
- Variable modifications of *Bacteroidetes*, a reduction in *Faecalibacterium* and *Coprococcus*, and an increase in *Eggertehella* [92].
- A decrease *Roseburia* [1,42,49,93,94].
- Low *Faecalibacterium* in AN patients with major depression [92].
- An increase in *Firmicutes* and *Ruminococcaceae* in AN patients and a decrease in cases with anxiety [42,51,95].

### 3.5. Gut Microbiota and BMI

Body mass index is considered a good indicator of intestinal dysbiosis and metabolic alterations, being also considered an index of AN severity. Unlike Di Lodovico et al. (2021), after analysing several studies, we concluded that *Roseburia* correlates with BMI value. The same author observed increased levels of *Clostridium*, *Akermansia*, and *Eisenbergiella* in AN patients [94]. In their study, Million M. et al. (2013) observed that *M. smithii* has an increased level in patients with a BMI under 25 kg/m^2^. It also concluded that the percentage of some bacteria in the microbiome is directly proportional to BMI. Thus, the lower the BMI, the higher the level of *Bifidobacterium animalis*, *M. smithii*, and *E. coli* species, while the *Lactobacillus* level is lower (Table 2) [45]. Morkl et al. (2017) found a negative correlation between BMI and *Bacteroides uniformis* (Table 2) [44].

Furthermore, in the AN group, both weight, and BMI showed statistically significant negative correlations with the abundance of bacteroidota and *Bacteroides*, but a positive correlation with *Subdoligranulum*. Additionally, BMI demonstrated a significant positive correlation with *Firmicutes*. These associations provide valuable insights into potential relationships between gut microbiota composition and clinical indicators, such as weight, BMI, and microbial taxa, in individuals with AN [43].

### 3.6. Gut Microbiota after Weight Gain

Understanding the mechanisms by which the brain–gut microbiota influences AN is important since AN is a multifactorial disease, which can influence genetic predispositions. The change in the intestinal microbiota associated with the improvement of the clinical picture in patients with AN and anxiety or depression is interesting, but it cannot be established whether the change represents the cause or the effect [2].

The composition of the human microbiome depends on the diets followed in the long term, but the microbiome can change rapidly in 3–4 days following short-term changes in the diet, being at the same time reversible [96]. According to Devkota et al. (2012) and David et al. (2014), changes in the composition of the microbiome can occur even after only one day after changing the diet, returning to the previous composition two days after the cessation of the new diet [97,98].

During hospitalization, refeeding affects the composition of the gut microbiota, but after the hospitalization the patients with AN receive a diet rich in calories, fat, and carbohydrates [5]. Also, some intestinal microbes like *Bacteroides*, *Lactobacillus*, *Helicobacter pylori*, *Escherichia coli*, and *Candida* contain proteins that have amino acid sequences identical to the appetite-regulating peptides [99]. The efficacy of nutritional treatment and successful weight restoration in individuals diagnosed with AN did not lead to a complete normalization of their gut microbiota composition in comparison to non-AN comparison control groups. These limited findings suggest that AN’s pathophysiology, particularly concerning the gut microbiome, may persist even after achieving weight restoration [66].

After the re-fed of patients with AN, the microbiome diversity increased but remained lower compared to healthy individuals [100]. High caloric and high fat diets used post-rehabilitation contribute at the increase in *Firmicutes* and decrease in *Bacteroidetes* [66].

At baseline, Mack et al. (2016) found decreased levels of *Verrumcomicrobia* and *Bifidobacteria*, and the *Bacteroidetes/Firmicutes* ratio remained reduced both before and after weight gain (Table 2) [42].

Kleiman et al. (2015) observed differences in the composition of the intestinal microbiota in patients with AN both at the beginning of treatment and after weight gain compared to healthy subjects. Also, using the Beck depression inventory and the Beck anxiety inventory, they observed a correlation between the increase in the number and diversity of the microbiota and the well-being of patients with AN. Improving the composition of the microbiota can have effects on reducing the severity of symptoms, normalizing weight, and improving the general condition of these patients. In the study of the same author, the lower abundance of *Bacteroidetes* persists after weight restoration, but *Firmicutes* increased (Table 2) [40].

Also, as other studies said, after weight gain, patients with AN presented:

- An increase in *Firmicutes*, *Ruminococcaceae*, *Faecalibacterium*, *Fusicatenibacter*, and *Lachnospiraceae*.
- A reduction in *Bacteroides* and *Parabacteroides*.
- An increase in *Leuconostocaceae* and a decrease in *Collinsella*, *Parabacteroides*, and *Catabacter* [101].
- The non-modification of the *Roseburia* species [93].
- A higher abundance of *Roseburia*, *Ruminococcus*, and *Faecalibacterium* species [94].

According to Vaher et al. (2022), weight recovery is associated with the re-establishment of the *Firmicutes*/*Bacteroidetes* ratio [31].

A diet with animal products used for nutrition can stimulate the growth of some bacteria that can be trigger factors for inflammation [24,40]. The supplementation with omega-3 polyunsaturated fatty acids like docosahexaenoic acid (DHA) reduces anxiety and depressive behaviours [102]. Moreover, omega-3 acids modulate the composition and normalize the function of the gut microbiota [103]. In animal studies, it was observed that the ingestion of saturated fatty acids leads to the growth of *Bilophila wadsworthia*, a growth associated with inflammatory bowel disease [97,98]. According to Herpertz-Dahlmann et al. (2017), long-term diets, as well as malnutrition, can have an impact on the gut microbiota and the brain [40].

Fermented food influences the composition of gut microbiota and has beneficial effects on mental health. Therefore, fermented food can be considered for some psychiatric disorders, including AN, with some authors proposing their addition to nutritional treatment protocols [82].

Animal studies have revealed that the microbiota play a role in the regulation of body mass and appetite in AN. Positive correlations were found between the level of *Bifidobacterium* and *Lactobacillus* and the plasma level of leptin, and negative correlations were found between *Clostridium*, *Bacteroides*, and *Prevotella* and the plasma level of leptin, while the plasma level of ghrelin was positively correlated with the level of *Bacteroides* and *Prevotella* and negative with *Bifidobacterium*, *Lactobacillus*, *Blautia coccoides*, and *Enterobacterium rectale* [32].

### 3.7. Therapy

Promising therapies using probiotics, prebiotics, faecal transplantation, and precision nutrition are being discussed today [75,104]. Modulating the intestinal microbiota with probiotics may be useful in various diseases involving the gut–brain–microbiota axis [31]. Treatment with probiotics relieves anxiety, a fact demonstrated by studies conducted both on humans and animals [91]. Although probiotics and prebiotics are effective in the treatment of depression, and anxiety and improve cognitive function, there are not many studies supporting their use in AN [105].

In their study on 22 patients with AN, Specht et al. (2022) observed an inverse relationship between changes in proinflammatory cytokines and specific microbial taxa possessing well-documented anti-inflammatory properties, such as *Lachnospiraceae* and *Anaerostipes*. Notably, the increased abundance of *Lachnospiraceae* during short-term weight recovery could potentially contribute to the reduction in TNF-α levels [106]. Previous research has linked *Lachnospiraceae* to favourable effects on cardiovascular risk factors and anti-inflammatory effects in the context of inflammatory bowel disease [107,108]. Moreover, Schulz et al. (2021) identified a significant association between higher *Lachnospiraceae* levels upon admission and shorter durations of treatment, a well-known indicator of a positive treatment outcome, even after accounting for other established predictors [95].

Conversely, *Dialister*, another microbial genus, has been reported to correlate with pro-inflammatory disease activity in spondyloarthritis. *Dialister* belongs to the family *Veillonaceae*, which has been linked to pro-inflammatory events and increased inflammation in individuals with cirrhosis. These findings collectively suggest the potential for a bidirectional interdependence between gut microbiota and inflammation in individuals with AN. The observed associations between specific microbial taxa and proinflammatory processes may hold valuable insights into the intricate relationship between gut microbiota composition and inflammatory states in AN [106].

## 4. Discussion

Considering that most cases of AN begin in adolescence, the effects of hormonal changes that occur at puberty should also be taken into account. Studying the gut–brain axis, especially in adolescents with AN, may be important for the long-term prognosis of this disease.

Research on the relationship between gut microbiota and anorexia nervosa is still in its early stages, and findings are not yet conclusive. However, there is growing interest in understanding the potential role of the gut–brain axis in eating disorders, including anorexia nervosa.

Some studies have suggested that individuals with anorexia nervosa may have alterations in the composition of their gut microbiota. However, the specific bacterial species or imbalances in proportions are not consistently reported across studies, and more research is needed to establish clear patterns. Significantly, conducting extensive research on the gut microbiome in this population holds the potential to augment our comprehension of disordered eating behaviour, dysregulated appetite, and the occurrence of comorbid depression and anxiety. This knowledge could ultimately contribute to the formulation of more effective treatment approaches and therapeutic strategies for individuals afflicted by AN.

The role of microbiota in AN has garnered significant attention in recent research. Several previously presented studies have highlighted potential connections between alterations in the gut microbiota and AN pathophysiology. While the precise mechanisms remain to be fully elucidated, emerging evidence suggests that the gut microbiota might influence various aspects of AN, including appetite regulation, nutrient absorption, immune function, and even behavioural aspects related to eating behaviours and mood. The influence of gut microbiota on AN is a complex and multifaceted process that is still being actively researched. While the exact mechanisms are not fully understood, several potential pathways have been proposed based on emerging evidence.

The key concepts related to the role of the gut microbiota in anorexia nervosa can be: the composition of gut microbiota, the neurotransmitter influence (serotonin production, Gamma-Aminobutyric acid (GABA) production, dopamine metabolism, norepinephrine and epinephrine production, SCFAs, vagus nerve stimulation, modulation of neurotransmitter receptors, influence on neuroinflammation), immune system modulation (microbial composition and immune function—immune system modulation SCFAs, tight junction integrity, immune cell activation, regulation of inflammation, dysbiosis and chronic inflammation, cytokine release, vagus nerve communication, feedback loop of chronic inflammation influencing the gut microbiota and vice versa, microbial metabolism, hormonal regulation, ghrelin regulation, leptin regulation, dietary input, appetite and metabolism regulation, brain response, impact on body weight), hormonal regulation (production of SCFAs, gut–brain axis communication, regulation of ghrelin, modulation of leptin, impact on insulin sensitivity, metabolism of bile acids, influence on peptide hormones, effects of gut microbial metabolites), nutrient absorption, psychological factors, and dietary habits [64,65].

Studies have identified differences in the gut microbiota composition of individuals with AN compared to healthy controls. These alterations may impact the metabolic and signalling pathways, affecting food intake and energy homeostasis. Analysis of the microbiota through the faeces currently remains the most convenient method, being non-invasive.

The gut–brain axis is a bidirectional communication system that involves signalling between the gut and the central nervous system. The gut microbiota can influence this axis by producing neuroactive compounds and metabolites that can influence brain function and behaviour.

It is important to note that the field of research on the gut microbiota and its impact on mental health, including eating disorders, is still evolving. Factors such as diet, stress, and other environmental influences can also play a role in shaping the composition of the gut microbiota. Additionally, causation and directionality of the relationship between gut microbiota and anorexia nervosa are not fully understood.

Dysbiosis, characterised by an imbalance in the gut microbiota, has been implicated in the pathogenesis of anorexia nervosa. This may play a role in the persistence of the disorder and its associated symptoms. Dysbiosis in the gut microbiota can trigger an immune response, leading to inflammation. Chronic inflammation has been implicated in AN and may contribute to the disease’s pathogenesis.

Microbiota alterations may impact the immune and inflammatory responses in individuals with anorexia nervosa. These findings may provide insights into the underlying mechanisms of the disorder and its comorbidities.

The gut microbiota plays a role in appetite regulation by producing signals that affect hunger and satiety. Changes in gut microbiota composition may influence an individual’s eating behaviours and food preferences.

An altered gut microbiota can affect gut barrier integrity, leading to increased gut permeability. This phenomenon, known as “leaky gut”, may allow harmful substances to cross into the bloodstream, potentially influencing systemic inflammation and immune responses.

The gut microbiota can produce neurotransmitters and neuroactive compounds that may impact mood and behaviour. Changes in the gut microbiome could potentially influence the development and severity of eating disorders, including AN. The gut microbiota’s influence on brain function may also impact psychological and behavioural factors associated with AN, such as anxiety, depression, and disordered eating patterns.

The interplay between gut microbiota and psychological factors in the development and maintenance of anorexia nervosa warrants further investigation. Comprehensive treatment strategies should consider both biological and psychological aspects.

Altered gut microbiota can impact nutrient absorption and metabolism, potentially leading to malnutrition and other metabolic abnormalities observed in AN. For instance, the well-established effects of diets enriched with saturated fats or high in prebiotic fibre on gut microbial composition and subsequent physiological functions hold promise for informing the development of nutritional rehabilitation diets in AN.

The variability in microbiota profiles among individuals with anorexia nervosa highlights the need for personalized treatment approaches. Tailoring interventions based on an individual’s unique gut microbiota may enhance therapeutic outcomes.

The complexity of anorexia nervosa and its multifaceted aetiology requires interdisciplinary collaboration between gastroenterologists, psychiatrists, dietitians, and psychologists to address both the microbiota and psychological aspects comprehensively.

The intricate relationship among gut microbiota, physiological determinants, and the aetiology of anorexia nervosa constitutes a multifaceted and burgeoning area of investigation. The bidirectional communication between the gut and the brain, acknowledged as the gut–brain axis, encompasses mechanisms that can exert influence over both physiological and psychological facets of an individual. Although ongoing research has yet to conclusively establish findings in this domain, several plausible modalities through which these factors may interrelate merit consideration. The gut microbiota possesses the capacity to modulate the production of neurotransmitters, notably serotonin and gamma-aminobutyric acid (GABA), integral to mood regulation and implicated in mental health disorders, including anorexia nervosa. The participation of the gut microbiota in immune system modulation is evident, exerting influence over bodily inflammation. The dysregulation of the immune system and chronic inflammation have been implicated in the pathophysiology of diverse psychiatric disorders, encompassing eating disorders. The gut microbiota’s impact extends to the regulation of hormones associated with appetite and metabolism, such as ghrelin and leptin. Dysregulation in these hormonal systems may contribute to deviations in eating behaviours and body weight. Alterations in the composition of the gut microbiota can impinge upon nutrient absorption and energy metabolism, potentially influencing body weight dynamics and contributing to the genesis or perpetuation of anorexia nervosa. Psychological determinants, encompassing stress and mood disorders, can wield influence over the composition of the gut microbiota. Concurrently, the gut microbiota can generate bioactive molecules capable of impacting brain function and behaviour, thereby contributing to the psychological dimensions of anorexia nervosa. Dietary practices, characterised by the restrictive eating patterns prevalent in anorexia nervosa, have the capacity to mould the composition of the gut microbiota. Conversely, the gut microbiota can modulate food preferences and cravings, potentially influencing an individual’s eating comportment.

It Is Imperative to underscore that despite the presence of evidence substantiating the potential involvement of the gut microbiota in mental health, including eating disorders, the field is undergoing continual evolution, and the establishment of causation remains a formidable challenge. Genetic predisposition, environmental influences, and individual variabilities stand as concomitant determinants of significance in the intricate tapestry of anorexia nervosa development.

### Future Directions

Understanding the role of gut microbiota in AN may open up new avenues for treatment and intervention. Probiotics, prebiotics, or dietary modifications targeting the gut microbiome could be explored as potential therapeutic strategies.

Manipulating the gut microbiota through dietary interventions, probiotics, or other means could offer a novel approach to complement traditional treatments for anorexia nervosa in children. However, more research is required to validate the effectiveness and safety of such interventions.

Longitudinal studies tracking changes in the microbiota composition over time in children with anorexia nervosa are essential to better understand the dynamic nature of this relationship and the potential for microbiota-based markers of disease progression.

Further research is needed to establish causality and identify specific microbial signatures that contribute to AN’s development and progression and may be targeted for therapeutic interventions in AN. Nonetheless, understanding the gut microbiota’s potential role in AN may offer new avenues for therapeutic interventions and personalized treatment strategies in the future.

Future research should focus on elucidating the precise mechanisms linking microbiota alterations and anorexia nervosa in children. Additionally, investigating the potential of microbiota-based interventions and their long-term effects should be a priority in advancing our understanding and treatment of this complex disorder.

## 5. Conclusions

The existing body of literature suggests a potential association between altered microbiota composition and anorexia nervosa in children. However, the research in this area remains limited, and further studies are needed to confirm and expand upon these findings.

While the field of microbiota research in AN is still evolving, it holds promise as a novel approach to better understanding the complex interplay between gut microbiota and the pathogenesis of AN. In the interim, if these mechanisms are proposed based on current research, it is essential to note that the relationship between gut microbiota and AN is still an active area of investigation.

## Figures and Tables

**Table 1:** Prevalence of AN.

No.	Study	Author	Year	Lot	Prevalence	Reference
1	Assessing disorders in children	Grigoroiu Ṣerbănescu	1987	8–11 years	0.01% girls; 0% boys	[9]
2	Incidence of AN	Hoeck et al.	2003		0.01%	[10]
3	Prevalence of eating disorders in secondary school students	Kovacs Krizbai	2009	secondary school students	0.2% girls	[11]
4	Current trends in epidemiology of eating disorders (including AN)	Herpertz-Dahlmann et al.	2009	12–18 years	1–4%	[12]
5	Prevalence of eating disorders in adolescents	Swanson et al.	2011	13–18 years	0.30%	[13]
6	Assessment of AN	Zipfel et al.	2015	males, females	1%	[14]
7	To assess the prevalence of mental disorders	Wagner et al.	2017	10–18 years	1.44%	[15]
8	To estimate the incidence of AN	Petkova et al.	2019	8–17 years	0.01%	[16]

**Table 2:** The examination of investigations conducted with the primary aim of elucidating the heterogeneity and microbial composition within the microbiota of individuals afflicted with AN.

Authors	Armougom et al. [41]	Mack et al. [42]	Yuan et al. [43]	Borgo et al. [1]	Morkl et al. [44]	Million et al. [45]	Kleiman et al. [46]	Kleiman et al. [47]	Morita et al. [48]
Year	2009	2016	2022	2017	2017	2013	2017	2015	2015
Study type	cross-sectional	longitudinal	cross-sectional	cross-sectional	cross-sectional	cross-sectional	longitudinal, case series	longitudinal	cross-sectional
Data analysis method		Chao index, Shannon index, Unifrac, Bray–Curtis	Mann–Whitney U test/Chao/Shannon/Sobs/Ace index/Adonis analysis		Chao index		Shannon index	Chao index, UniFrac distances	Kruskal–Wallis, Wilcoxon, Mann–Whitney
Study group size	9	44	30	15	18	15	3	16	25
Control group size tents)	20	55	30	15	20	76	absent	12	21
Age (years)	19–36	23.8 ± 6.8	16		22.44 ± 3.52	27.3 ± 10.8	23.3	28 ± 11.7	30 ± 10.2
Increased level	*Bacteroidetes*, *M. smithii*	*Firmicutes*, *Actinobacteria*, *Proteobacteria*, *Verrucomicrobia*, *Methanobrevibacter*, *Clostridium cluster XI*	*Lachnospiraceae*, *Streptococcaceae*, *Bacteroidaceae*, *Coriobacteriaceae*, *Rikenellaceae*, *Enterobacteriaceae*,	*Proteobacteria*, *Enterobacteriaaceae*	*Coriobacteriaceae* *Roseburia*, *M. smithii, Clostridium*	*Bacteroidetes*, *M. smithii*		*Coriobacteriales*, *Lactobacillales*	*Clostridioides difficile*
Decreased level	*Lactobacillus*	*Bacteroidetes*	*Ruminococcaceae*, *Bifidobacteriaceae*, *Peptostreptococcaceae*, *Oscillospiraceae*, *Burkholderiaceae*	*Firmicutes*, *Ruminococcaceae*, *Ruminococcus*, *Roseburia*, *Clostridium*				*Clostridia*, *Anaerostipes*, *Faecalibacterium*	*Clostridium coccoides*, *Clostridium leptum*, *Bacteroides fragilis*, *Streptococcus*, *Lactobacillus plantarum*
Type of dysbiosis	Alpha and beta	Alpha and beta	Only beta	Alpha and beta	Alpha and beta	Alpha and beta	Alpha and beta	Alpha and beta	Alpha and beta
Other associated biomarkers		elevated concentrations of branched-chain fatty acids		reduction in total short-chain fatty acids, butyrate, propionate					lower CRP, higher ASAT and ALAT
Sample	faeces	faeces	faeces	faeces	faeces	faeces	faeces	faeces	faeces

**Table 3:** Dietary profile vs. gut microbiota in AN.

Authors	Study	Year	Results	Reference
Palmnäs et al.	How aspartame influences gut microbial composition	2014	Aspartame consumption correlates with an increase in the prevalence of *Enterobacteriaceae*	[71]
Suez et al.	Consumption of non-caloric artificial sweetners inducts compositional and functional alterations to the intestinal microbiota.	2014	Existence of a connection between the use of non-caloric artificial sweeteners and the proliferation of *Bacteroides vulgatus*, *Bacteroides fragilis*, *Akkermansia muciniphyla*, *Lactobacillus reuteri*, as well as alterations in the *Bacteroides/Firmicutes* ratio	[72]
Glick-Bauer et al.	The Health Advantage of a Vegan Diet: Exploring the Gut Microbiota Connection	2014	The prevalence of *Prevotella* spp. and a decrease in *Bacteroides* in vegetarians	[73]
Borgo et al.	Microbiota in AN	2017	The reduction in *Roseburia* species, particularly *Roseburia inulinivorans*, is associated with diminished propionate production, and the concentrations of butyrate and *Clostridium* spp. are inversely correlated with the severity of anxiety and depression	[1]
Herpertz-Dahlmann et al.	Composition of the diet influences gut microbiota	2017	Low carbohydrate and low-fat dietary regimens are associated with increased levels of *Firmicutes* and *Proteobacteria*, accompanied by decreased levels of *Bacteroidetes*.	[40]
Mendez-Figueroa et al.	Influence of food intake on host-gut microbiota in AN	2019	*Bifidobacterium*, *Bacteroides*, and *Verrucomicrobia*, which metabolize non-digestible carbohydrates, exhibit altered dynamics in patients with AN	[74]
Kim et al.	Diet composition and gut microbiota	2020	Fruits containing polysaccharides correlate with increased *Bifidobacterium* levels, while vegetable intake exhibits inverse associations with *Proteobacteria* and *Thermi* populations.	[68]
Berding et al.	Diet as a major factor involved in shaping the gut microbiota composition	2021	Dietary patterns centred on fruits, vegetables, and vegetarian regimes contribute to enhanced microbial flora diversity, with a predominant representation of *Bacteroidetes* and *Actinobacteria* species. Augmented fibre intake is associated with heightened microbiota diversity and elevated levels of beneficial bacteria, including Bifidobacterium, Lactobacillus, Bacteroides, Roseburia, and Prevotella.	[30]
			Fruits containing polysaccharides correlate with increased *Bifidobacterium* levels, while vegetable intake exhibits inverse associations with *Proteobacteria* and *Thermi* populations.	[30]
			Consumption of whole-grain fibre is linked to an increased abundance of *Actinobacteria*, *Bifidobacterium*, *Clostridium*, *Akkermansia*, and *Roseburia*	[30]
			Polyphenols contribute to the augmentation of bifidobacteria and lactobacilli while decreasing the presence of *Clostridium perfringens* and *C. histolyticum* species.	[30]
Andrioaie et al.	Influence of diet in psychiatric disorders	2022	An increase in both *Prevotella* and *Bacteroides* in vegetarians	[75]

## References

[1] Borgo F., Riva A., Benetti A., Casiraghi M.C., Bertelli S., Garbossa S., Anselmetti S., Scarone S., Pontiroli A.E., Morace G. (2017). Microbiota in anorexia nervosa: The triangle between bacterial species, metabolites and psychological tests. PLoS ONE.

[2] Garcia-Gil M., Ceccarini M.R., Stoppini F., Cataldi S., Mazzeschi C., Delvecchio E., Albi E., Gizzi G. (2022). Brain and gut microbiota disorders in the psychopathology of anorexia nervosa. Transl. Neurosci..

[3] Dhopatkar N., Keeler J.L., Mutwalli H., Whelan K., Treasure J., Himmerich H. (2023). Gastrointestinal symptoms, gut microbiome, probiotics and prebiotics in anorexia nervosa: A review of mechanistic rationale and clinical evidence. Psychoneuroendocrinology.

[4] Seitz J., Belheouane M., Schulz N., Dempfle A., Baines J.F., Herpertz-Dahlmann B. (2019). The Impact of Starvation on the Microbiome and Gut-Brain Interaction in Anorexia Nervosa. Front. Endocrinol..

[5] Breton J., Dechelotte P., Ribet D. (2019). Intestinal microbiota and Anorexia Nervosa. Clin. Nutr. Exp..

[6] van der Gun L.L. (2023). Elucidating the Role of the Anorectic Gut Microbiome in Value-Based Decision Making and Reward Signalling. Master’s Thesis.

[7] Flint H.J. (2011). Obesity and the gut microbiota. J. Clin. Gastroenterol..

[8] Hornberger L.L., Lane M.A., Committee on adolescence (2021). Identification and Management of Eating Disorders in Children and Adolescents. Pediatrics.

[9] Grigoroiu-Şerbănescu M.A. (1987). Brief inventory for assessing personality traits and disorders in children aged 8–11. Rev. Roum. Neurol. Psychiatr..

[10] Hoeck H.W., van Hoeken D. (2003). Review of the prevalence and incidence of eating disorders. Int. J. Eat. Disord..

[11] Kovács Krizbai T., Szabó P. (2009). Prevalence of eating disorders in Romanian, Hungarian and Saxon secondary school students in Transylvania. Psychiatr. Hung..

[12] Herpertz-Dahlmann B. (2009). Adolescent Eating Disorders: Definitions, Symptomatology, Epidemiology and Comorbidity. Child Adolesc. Psychiatr. Clin. N. Am..

[13] Swanson S.A., Crow S.J., Le Grange D., Swendsen J., Merikangas K.R. (2011). Prevalence and correlates of eating disorders in adolescents. Results from the national comorbidity survey replication adolescent supplement. Arch. Gen. Psychiatry.

[14] Zipfel S., Giel K.E., Bulik C.M., Hay P., Schmidt U. (2015). Anorexia nervosa: Aetiology, assessment, and treatment. Lancet Psychiatry.

[15] Wagner G., Zeiler M., Waldherr K., Philipp J., Truttmann S., Dür W., Treasure J.L., Karwautz A.F.K. (2017). Mental health problems in Austrian adolescents: A nationwide, two-stage epidemiological study applying DSM-5 criteria. Eur. Child. Adolesc. Psychiatry.

[16] Petkova H., Simic M., Nicholls D., Ford T., Prina A.M., Stuart R., Livingstone N., Kelly G., Macdonald G., Eisler I. (2019). Incidence of anorexia nervosa in young people in the UK and Ireland: A national surveillance study. BMJ Open.

[17] Langdon-Daly J., Serpell L. (2017). Protective factors against disordered eating in family systems: A systematic review of research. J. Eat. Disord..

[18] Neale J., Hudson L.D. (2020). Anorexia nervosa in adolescents. Br. J. Hosp. Med..

[19] Argyrides M., Anastasiades E., Alexiou E. (2020). Risk and Protective Factors of Disordered Eating in Adolescents Based on Gender and Body Mass Index. Int. J. Environ. Res. Public Health.

[20] Barakat S., McLean S.A., Bryant E., Le A., Marks P., Touyz S., Maguire S. (2023). Risk factors for eating disorders: Findings from a rapid review. J. Eat. Disord..

[21] Freimer D., Yang T.T., Ho T.C., Tymofiyeva O., Leung C. (2022). The gut microbiota, HPA axis, and brain in adolescent-onset depression: Probiotics as a novel treatment. Brain Behav. Immun.-Health.

[22] Gorwood P., Blanchet-Collet C., Chartrel N., Duclos J., Dechelotte P., Hanachi M., Fetissov S., Godart N., Melchior J.C., Ramoz N. (2016). New Insights in Anorexia Nervosa. Front. Neurosci..

[23] Santonicola A., Gagliardi M., Guarino M.P.L., Siniscalchi M., Ciacci C., Iovino P. (2019). Eating disorders and gastrointestinal diseases. Nutrients.

[24] Schwensen H.F., Kan C., Treasure J., Høiby N., Sjögren M. (2018). A systematic review of studies on the faecal microbiota in anorexia nervosa: Future research may need to include microbiota from the small intestine. Eat. Weight Disord. EWD.

[25] Cryan J.F., Dinan T.G. (2012). Mind-altering microorganisms: The impact of the gut microbiota on brain and behaviour. Nat. Rev. Neurosci..

[26] Cox L.M., Blaser M.J. (2013). Pathways in microbe-induced obesity. Cell Metab..

[27] Foster J.A., McVey Neufeld K.A. (2013). Gut-brain axis: How the microbiome influences anxiety and depression. Trends Neurosci..

[28] Sarkar A., Harty S., Lehto S.M., Moeller A.H., Dinan T.G., Dunbar R.I.M., Cryan J.F., Burnet P.W.J. (2018). The Microbiome in Psychology and Cognitive Neuroscience. Trends Cogn. Sci..

[29] Brett B.E., de Weerth C. (2019). The microbiota-gut-brain axis: A promising avenue to foster healthy developmental outcomes. Dev. Psychobiol..

[30] Berding K., Vlckova K., Marx W., Schellekens H., Stanton C., Clarke G., Jacka F., Dinan T.G., Cryan J.F. (2021). Diet and the Microbiota-Gut-Brain Axis: Sowing the Seeds of Good Mental Health. Adv. Nutr..

[31] Vaher K., Bogaert D., Richardson H., Boardman J.P. (2022). Microbiome-gut-brain axis in brain development, cognition and behaviour during infancy and early childhood. Dev. Rev..

[32] Karakuła-Juchnowicz H., Pankowicz H., Juchnowicz D., Valverde Piedra J.L., Małecka-Massalska T. (2017). Intestinal microbiota—A key to understanding the pathophysiology of anorexia nervosa?. Psychiatr. Pol..

[33] Terry S.M., Barnett J.A., Gibson D.L. (2022). A critical analysis of eating disorders and the gut microbiome. J. Eat. Disord..

[34] O’Mahony S.M., Marchesi J.R., Scully P., Codling C., Ceolho A.M., Quigley E.M., Cryan J.F., Dinan T.G. (2009). Early life stress alters behaviour, immunity, and microbiota in rats: Implications for irritable bowel syndrome and psychiatric illnesses. Biol. Psychiatry.

[35] Allen R.G., Lafuse W.P., Galley J.D., Ali M.M., Ahmer B.M., Bailey M.T. (2012). The intestinal microbiota is necessary for stressor-induced enhancement of splenic macrophage microbicidal activity. Brain Behav. Immun..

[36] Gareau M.G., Silva M.A., Perdue M.H. (2008). Pathophysiological mechanisms of stress-induced intestinal damage. Curr. Mol. Med..

[37] Ait-Belgnaoui A., Durand H., Cartier C., Chaumaz G., Eutamene H., Ferrier L., Houdeau E., Fioramonti J., Bueno L., Theodorou V. (2012). Prevention of gut leakiness by a probiotic treatment leads to attenuated HPA response to acute psychological stress in rats. Psychoneuroendocrinology.

[38] Petra A.I., Panagiotidou S., Stewart J.M., Conti P., Theoharides T.C. (2014). Spectrum of mast cell activation disorders. Expert Rev. Clin. Immunol..

[39] Socała K., Doboszewska U., Szopa A., Serefko A., Włodarczyk M., Zielińska A., Poleszak E., Fichna J., Wlaź P. (2021). The role of microbiota-gut-brain axis in neuropsychiatric and neurological disorders. Pharmacol. Res..

[40] Herpertz-Dahlmann B., Seitz J., Baines J. (2017). Food matters: How the microbiome and gut-brain interaction might impact the development and course of anorexia nervosa. Eur. Child Adolesc. Psychiatry.

[41] Armougom F., Henry M., Vialettes B., Raccah D., Raoult D. (2009). Monitoring bacterial community of human gut microbiota reveals an increase in Lactobacillus in obese patients and Methanogens in anorexic patients. PLoS ONE.

[42] Mack I., Cuntz U., Grämer C., Niedermaier S., Pohl C., Schwiertz A., Zimmermann K., Zipfel S., Enck P., Penders J. (2016). Weight gain in anorexia nervosa does not ameliorate the faecal microbiota, branched-chain fatty acid profiles, and gastrointestinal complaints. Sci. Rep..

[43] Yuan R., Yang L., Yao G., Geng S., Ge Q., Bo S., Li X. (2022). Features of gut microbiota in patients with anorexia nervosa. Chin. Med. J..

[44] Morkl S., Lackner S., Müller W., Gorkiewicz G., Kashofer K., Oberascher A., Painold A., Holl A., Holzer P., Meinitzer A. (2017). Gut microbiota and body composition in anorexia nervosa inpatients in comparison to athletes, overweight, obese, and normal weight controls. Int. J. Eat. Disord..

[45] Million M., Angelakis E., Maraninchi M., Henry M., Giorgi R., Valero R., Vialettes B., Raoult D. (2013). Correlation between body mass index and gut concentrations of *Lactobacillus reuteri*, *Bifidobacterium animalis*, *Methanobrevibacter smithii* and *Escherichia coli*. Int. J. Obes..

[46] Kleiman S.C., Glenny E.M., Bulik-Sullivan E.C., Huh E.Y., Tsilimigras M.C.B., Fodor A.A., Bulik C.M., Carroll I.M. (2017). Daily Changes in Composition and Diversity of the Intestinal Microbiota in Patients with Anorexia Nervosa: A Series of Three Cases. Eur. Eat. Disord. Rev..

[47] Kleiman S.C., Watson H.J., Bulik-Sullivan E.C., Huh E.Y., Tarantino L.M., Bulik C.M., Carroll I.M. (2015). The Intestinal Microbiota in Acute Anorexia Nervosa and During Renourishment: Relationship to Depression, Anxiety, and Eating Disorder Psychopathology. Psychosom. Med..

[48] Morita C., Tsuji H., Hata T., Gondo M., Takakura S., Kawai K., Yoshihara K., Ogata K., Nomoto K., Miyazaki K. (2015). Gut Dysbiosis in Patients with Anorexia Nervosa. PLoS ONE.

[49] Hanachi M., Manichanh C., Schoenenberger A., Pascal V., Levenez F., Cournède N., Doré J., Melchior J.C. (2019). Altered host-gut microbes symbiosis in severely malnourished anorexia nervosa (AN) patients undergoing enteral nutrition: An explicative factor of functional intestinal disorders?. Clin. Nutr..

[50] Hata T., Miyata N., Takakura S., Yoshihara K., Asano Y., Kimura-Todani T., Yamashita M., Zhang X.T., Watanabe N., Mikami K. (2019). The Gut Microbiome Derived From Anorexia Nervosa Patients Impairs Weight Gain and Behavioral Performance in Female Mice. Endocrinology.

[51] Prochazkova P., Roubalova R., Dvorak J., Kreisinger J., Hill M., Tlaskalova-Hogenova H., Tomasova P., Pelantova H., Cermakova M., Kuzma M. (2021). The intestinal microbiota and metabolites in patients with anorexia nervosa. Gut Microbes.

[52] Roubalova R., Prochazkova P., Papezova H., Smitka K., Bilej M., Tlaskalova-Hogenov H. (2020). Anorexia nervosa: Gut microbiota-immune-brain interactions. Clin. Nutr..

[53] Jiang H., Ling Z., Zhang Y., Mao H., Ma Z., Yin Y., Wang W., Tang W., Tan Z., Shi J. (2015). Altered faecal microbiota composition in patients with major depressive disorder. Brain Behav. Immun..

[54] Lach G., Schellekens H., Dinan T.G., Cryan J.F. (2018). Anxiety, Depression, and the Microbiome: A Role for Gut Peptides. NeuroTherapeutics.

[55] Yang Z., Li J., Gui X., Shi X., Bao Z., Han H., Li M.D. (2020). Updated review of research on the gut microbiota and their relation to depression in animals and human beings. Mol. Psychiatry.

[56] Jiang H.Y., Zhang X., Yu Z.H., Zhang Z., Deng M., Zhao J.H., Ruan B. (2018). Altered gut microbiota profile in patients with generalized anxiety disorder. J. Psychiatr. Res..

[57] Zmora N., Suez J., Elinav E. (2019). You are what you eat: Diet, health and the gut microbiota. Nat. Rev. Gastroenterol. Hepatol..

[58] Xia X., He S.Y., Zhang X.L., Wang D., He Q., Xiao Q.A., Yang Y. (2023). The causality between gut microbiome and anorexia nervosa: A Mendelian randomization analysis. Front. Microbiol..

[59] Calcaterra V., Rossi V., Massini G., Regalbuto C., Hruby C., Panelli S., Bandi C., Zuccotti G. (2022). Precocious puberty and microbiota: The role of the sex hormone–gut microbiome axis. Front. Endocrinol..

[60] del Castillo-Izquierdo Á., Mayneris-Perxachs J., Fernández-Rea J.M. (2022). Bidirectional relationships between the gut microbiome and sexual traits. Am. J. Physiol. Cell Physiol..

[61] Tennoune N., Chan P., Breton J., Legrand R., Chabane Y.N., Akkermann K., Järv A., Ouelaa W., Takagi K., Ghouzali I. (2014). Bacterial ClpB heat-shock protein, an antigen-mimetic of the anorexigenic peptide α-MSH, at the origin of eating disorders. Transl. Psychiatry.

[62] Sinno M.H., Do Rego J.C., Coëffier M., Bole-Feysot C., Ducrotté P., Gilbert D., Tron F., Costentin J., Hökfelt T., Déchelotte P. (2009). Regulation of feeding and anxiety by alpha-MSH reactive autoantibodies. Psychoneuroendocrinology.

[63] Breton J., Legrand R., Akkermann K., Järv A., Harro J., Déchelotte P., Fetissov S.O. (2016). Elevated plasma concentrations of bacterial ClpB protein in patients with eating disorders. Int. J. Eat. Disord..

[64] Glenny E.M., Bulik-Sullivan E.C., Tang Q., Bulik C.M., Carroll I.M. (2017). Eating Disorders and the Intestinal Microbiota: Mechanisms of Energy Homeostasis and Behavioral Influence. Curr. Psychiatry Rep..

[65] Ghenciulescu A., Park R.J., Burnet P.W.J. (2021). The Gut Microbiome in Anorexia Nervosa: Friend or Foe?. Front. Psychiatry.

[66] Ruusunen A., Rocks T., Jacka F., Loughman A. (2019). The gut microbiome in anorexia nervosa: Relevance for nutritional rehabilitation. Psychopharmacology.

[67] Gröbner E.M., Zeiler M., Fischmeister F.P.S., Kollndorfer K., Schmelz S., Schneider A., Haid-Stecher N., Sevecke K., Wagner G., Keller L. (2022). The effects of probiotics administration on the gut microbiome in adolescents with anorexia nervosa—A study protocol for a longitudinal, double-blind, randomized, placebo-controlled trial. Eur. Eat. Disord. Rev..

[68] Kim Y.S., Unno T., Kim B.Y., Park M.S. (2020). Sex Differences in Gut Microbiota. World J. Mens Health.

[69] Chakrabarti A., Geurts L., Hoyles L., Iozzo P., Kraneveld A.D., La Fata G., Miani M., Patterson E., Pot B., Shortt C. (2022). The microbiota–gut–brain axis: Pathways to better brain health. Perspectives on what we know, what we need to investigate and how to put knowledge into practice. Cell. Mol. Life Sci..

[70] Margolis K.G., Cryan J.F., Mayer E.A. (2021). The Microbiota-Gut-Brain Axis: From Motility to Mood. Gastroenterology.

[71] Palmnä M.S.A., Cowan T.E., Bomhof M.R., Su J., Reimer R.A., Vogel H.J., Hittel D.S., Shearer J. (2014). Low-Dose Aspartame Consumption Differentially Affects Gut Microbiota-Host Metabolic Interactions in the Diet-Induced Obese Rat. PLoS ONE.

[72] Suez J., Korem T., Zeevi D., Zilberman-Schapira G., Thaiss C.A., Maza O., Israeli D., Zmora N., Gilad S., Weinberger A. (2014). Artificial sweeteners induce glucose intolerance by altering the gut microbiota. Nature.

[73] Glick-Bauer M., Yeh M.C. (2014). The Health Advantage of a Vegan Diet: Exploring the Gut Microbiota Connection. Nutrients.

[74] Mendez-Figueroa V., Biscaia J.M., Mohedano R.B., Blanco-Fernandez A., Bailen M., Bressa C., Larrosa M., Gonzalez-Soltero R. (2019). Can Gut Microbiota and Lifestyle Help Us in the Handling of Anorexia Nervosa Patients?. Microorganisms.

[75] Andrioaie I.M., Duhaniuc A., Nastase E.V., Iancu L.S., Lunca C., Trofin F., Anton-Păduraru D.T., Dorneanu O.S. (2022). The Role of the Gut Microbiome in Psychiatric Disorders. Microorganisms.

[76] Misra M., Tsai P., Anderson E.J., Hubbard J.L., Gallagher K., Soyka L.A., Miller K.K., Herzog D.B., Klibanski A. (2006). Nutrient intake in community-dwelling adolescent girls with anorexia nervosa and in healthy adolescents. Am. J. Clin. Nutr..

[77] Chiurazzi C., Cioffi I., De Caprio C., De Filippo E., Marra M., Sammarco R., Di Guglielmo M.L., Contaldo F., Pasanisi F. (2017). Adequacy of nutrient intake in women with restrictive anorexia nervosa. Nutrition.

[78] Patsalos O., Dalton B., Kyprianou C., Firth J., Shivappa N., Hébert J.R., Schmidt U., Himmerich H. (2021). Nutrient Intake and Dietary Inflammatory Potential in Current and Recovered Anorexia Nervosa. Nutrients.

[79] Ayton A.K. (2004). Dietary polyunsaturated fatty acids and anorexia nervosa: Is there a link?. Nutr. Neurosci..

[80] Matzkin V.B., Geissler C., Coniglio R., Selles J., Bello M. (2006). Cholesterol concentrations in patients with Anorexia Nervosa and in healthy controls. Int. J. Psychiatr. Nurs. Res..

[81] Rigaud D., Tallonneau I., Vergès B. (2009). Hypercholesterolaemia in anorexia nervosa: Frequency and changes during refeeding. Diabetes Metab..

[82] Roubalova R., Prochazkova P., Papezova H. (2022). Linking Anorexia Nervosa with the Gut Microbiota. A New Narrative. Eat. Disord..

[83] Ramirez J., Guarner F., Bustos Fernandez L., Maruy A., Sdepanian V.L., Cohen H. (2020). Antibiotics as Major Disruptors of Gut Microbiota. Front. Cell. Infect. Microbiol..

[84] Dahiya D., Nigam P.S. (2023). Antibiotic-Therapy-Induced Gut Dysbiosis Affecting Gut Microbiota-Brain Axis and Cognition: Restoration by Intake of Probiotics and Synbiotics. Int. J. Mol. Sci..

[85] Jernberg C., Löfmark S., Edlund C., Jansson J.K. (2007). Long-term ecological impacts of antibiotic administration on the human intestinal microbiota. ISME J..

[86] Löfmark S., Jernberg C., Jansson J.K., Edlund C. (2006). Clindamycin-induced enrichment and long-term persistence of resistant *Bacteroides* spp. and resistance genes. J. Antimicrob. Chemother..

[87] Rashid M.U., Zaura E., Buijs M.J., Keijser B.J., Crielaard W., Nord C.E., Weintraub A. (2015). Determining the Long-term Effect of Antibiotic Administration on the Human Normal Intestinal Microbiota Using Culture and Pyrosequencing Methods. Clin. Infect. Dis..

[88] Palleja A., Mikkelsen K.H., Forslund S.K., Kashani A., Allin K.H., Nielsen T., Hansen T.H., Liang S., Feng Q., Zhang C. (2018). Recovery of gut microbiota of healthy adults following antibiotic exposure. Nat. Microbiol..

[89] Kim A.H., Lee Y., Kim E., Ji S.C., Chung J.Y., Cho J.Y. (2021). Assessment of Oral Vancomycin-Induced Alterations in Gut Bacterial Microbiota and Metabolome of Healthy Men. Front. Cell. Infect. Microbiol..

[90] Simpson C.A., Diaz-Arteche C., Eliby D., Schwartz O.S., Simmons J.G., Cowan C.S.M. (2021). The gut microbiota in anxiety and depression—A systematic review. Clin. Psychol. Rev..

[91] Morais L.H., Schreiber H.L., Mazmanian S.K. (2021). The gut microbiota-brain axis in behaviour and brain disorders. Nat. Rev. Microbiol..

[92] Nikolova V.L., Smith M.R.B., Hall L.J., Cleare A.J., Stone J.M., Young A.H. (2021). Perturbations in Gut Microbiota Composition in Psychiatric Disorders: A Review and Meta-analysis. JAMA Psychiatry.

[93] Mondot S., Lachkar L., Doré J., Blottière H.M., Hanachi M. (2022). Roseburia, a decreased bacterial taxon in the gut microbiota of patients suffering from anorexia nervosa. Eur. J. Clin. Nutr..

[94] Di Lodovico L., Mondot S., Doré J., Mack I., Hanachi M., Gorwood P. (2021). Anorexia nervosa and gut microbiota: A systematic review and quantitative synthesis of pooled microbiological data. Prog. Neuro-Psychopharmacol. Biol. Psychiatry.

[95] Schulz N., Belheouane M., Dahmen B., Ruan V.A., Specht H.E., Dempfle A., Herpertz- Dahlmann B., Baines J.F., Seitz J. (2021). Gut microbiota alteration in adolescent anorexia nervosa does not normalize with short-term weight restoration. Int. J. Eat. Disord..

[96] Walker A.W., Ince J., Duncan S.H., Webster L.M., Holtrop G., Ze X., Brown D., Stares M.D., Scott P., Bergerat A. (2011). Dominant and diet-responsive groups of bacteria within the human colonic microbiota. ISME J..

[97] Devkota S., Wang Y., Musch M.W., Leone V., Fehlner-Peach H., Nadimpalli A., Antonopoulos D.A., Jabri B., Chang E.B. (2012). Dietary-fat-induced taurocholic acid promotes pathobiont expansion and colitis in Il10^−/−^ mice. Nature.

[98] David L.A., Maurice C.F., Carmody R.N., Gootenberg D.B., Button J.E., Wolfe B.E., Ling A.V., Devlin A.S., Varma Y., Fischbach M.A. (2014). Diet rapidly and reproducibly alters the human gut microbiome. Nature.

[99] Holzer P., Farzi A. (2014). Neuropeptides and the microbiota-gut-brain axis. Adv. Exp. Med. Biol..

[100] Faraj J., Takanti V., Tavakoli H.R. (2021). The Gut-Brain Axis: Literature Overview and Psychiatric Applications. Fed. Pract..

[101] Monteleone A.M., Troisi J., Fasano A., Dalle Grave R., Marciello F., Serena G., Calugi S., Scala G., Corrivetti G., Cascino G. (2021). Multi-omics data integration in anorexia nervosa patients before and after weight regain: A microbiome-metabolomics investigation. Clin. Nutr..

[102] Davis D.J., Hecht P.M., Jasarevic E., Beversdorf D.Q., Will M.J., Fritsche K., Gillespie C.H. (2017). Sex-specific effects of docosahexaenoic acid (DHA) on the microbiome and behaviour of socially-isolated mice. Brain Behav. Immun..

[103] Pusceddu M.M., El Aidy S., Crispie F., O’Sullivan O., Cotter P., Stanton C., Kelly P., Cryan J.F., Dinan T.G. (2015). N-3 Polyunsaturated Fatty Acids (PUFAs) Reverse the Impact of Early-Life Stress on the Gut Microbiota. PLoS ONE.

[104] Garcia N., Gutierrez E. (2023). Anorexia nervosa and microbiota: Systematic review and critical appraisal. Eat. Weight Disord. EWD.

[105] Sun L., Zhang H., Cao Y., Wang C., Zhao C., Wang H., Cui G., Wang M., Pan Y., Shi Y. (2019). Fluoxetine ameliorates dysbiosis in a depression model induced by chronic unpredicted mild stress in mice. Int. J. Med. Sci..

[106] Specht H.E., Mannig N., Belheouane M., Andreani N.A., Tenbrock K., Biemann R., Borucki K., Dahmen B., Dempfle A., Baines J.F. (2022). Lower serum levels of IL-1β and IL-6 cytokines in adolescents with anorexia nervosa and their association with gut microbiota in a longitudinal study. Front. Psychiatry.

[107] Tindall A.M., McLimans C.J., Petersen K.S., Kris-Etherton P.M., Lamendella R. (2020). Walnuts and Vegetable Oils Containing Oleic Acid Differentially Affect the Gut Microbiota and Associations with Cardiovascular Risk Factors: Follow-up of a Randomized, Controlled, Feeding Trial in Adults at Risk for Cardiovascular Disease. J. Nutr..

[108] Sun M., Wu W., Liu Z., Cong Y. (2017). Microbiota metabolite short chain fatty acids, GPCR, and inflammatory bowel diseases. J. Gastroenterol..

